# Soil Manganese Enrichment from Industrial Inputs: A Gastropod Perspective

**DOI:** 10.1371/journal.pone.0085384

**Published:** 2014-01-14

**Authors:** Despina-Maria Bordean, Dragos V. Nica, Monica Harmanescu, Ionut Banatean-Dunea, Iosif I. Gergen

**Affiliations:** 1 Banat’s University of Agricultural Sciences and Veterinary Medicine from Timisoara, Faculty of Food Processing Technology, Timisoara, Romania; 2 Banat’s University of Agricultural Sciences and Veterinary Medicine from Timisoara, Faculty of Animal Sciences and Biotechnologies, Timisoara, Romania; 3 Banat’s University of Agricultural Sciences and Veterinary Medicine from Timisoara, Faculty of Agriculture, Timisoara, Romania; CINVESTAV-IPN, Mexico

## Abstract

Manganese is one of the most abundant metal in natural environments and serves as an essential microelement for all living systems. However, the enrichment of soil with manganese resulting from industrial inputs may threaten terrestrial ecosystems. Several studies have demonstrated harmful effects of manganese exposure by cutaneous contact and/or by soil ingestion to a wide range of soil invertebrates. The link between soil manganese and land snails has never been made although these invertebrates routinely come in contact with the upper soil horizons through cutaneous contact, egg-laying, and feeding activities in soil. Therefore, we have investigated the direct transfer of manganese from soils to snails and assessed its toxicity at background concentrations in the soil. Juvenile *Cantareus aspersus* snails were caged under semi-field conditions and exposed first, for a period of 30 days, to a series of soil manganese concentrations, and then, for a second period of 30 days, to soils with higher manganese concentrations. Manganese levels were measured in the snail hepatopancreas, foot, and shell. The snail survival and shell growth were used to assess the lethal and sublethal effects of manganese exposure. The transfer of manganese from soil to snails occurred independently of food ingestion, but had no consistent effect on either the snail survival or shell growth. The hepatopancreas was the best biomarker of manganese exposure, whereas the shell did not serve as a long-term sink for this metal. The kinetics of manganese retention in the hepatopancreas of snails previously exposed to manganese-spiked soils was significantly influenced by a new exposure event. The results of this study reveal the importance of land snails for manganese cycling in terrestrial biotopes and suggest that the direct transfer from soils to snails should be considered when precisely assessing the impact of anthropogenic Mn releases on soil ecosystems.

## Introduction

Manganese (Mn) is the fourth most abundant metal in natural environments and comprises about 0.1% of the Earth’s crust [1]. As a result of being actively assimilated and used by both plants and animals [2], this trace element is important to ecosystem functioning. The essentiality of Mn for living systems results from the fact that this metal is an essential cofactor for several classes of enzymes, such as oxidoreductases, transferases, ligases, or hydrolases [3]. Relevant examples of Mn-based enzymes that occur in animals are manganese superoxide dismutase (MnSOD), which catalyzes the dismutation of two molecules of superoxide anion into water and hydrogen peroxide, and arginase (Arg), which catalyzes the hydrolysis of.

L-arginine to produce L-ornithine and urea [4]–[6]. However, manganese can pose serious threats to both aquatic and terrestrial ecosystems [2], [5], [7]. As environmental problems become global in scope, manganese contamination originating from escalating mining, manufacturing, agricultural, and industrial activities, especially in the less developed countries, has raised serious concerns about its putative ecological side-effects. As a result, manganese is currently regarded as a new emerging contaminant in the environment [5], and its toxicity has attracted considerable scientific interest from ecotoxicologists and environmental chemists.

Manganese exists in soils both in organic and inorganic forms, in several oxidation states, i.e., 0, +2, +3, +4, +6, +7 [8]. Research emphasis has been placed on the toxic effects of compounds containing inorganic Mn^2+^, Mn^3+^, and Mn^4+^ ions since these are the forms most commonly encountered in biological systems [9]. Most studies have addressed the accumulation of Mn in animals via food uptake [2], and those which addressed the direct uptake from soil by cutaneous contact and/or by soil ingestion have generally used earthworms, nematodes, or collembolans as an invertebrate study system [10]–[12]. Such studies were conducted under controlled laboratory environments, in which test invertebrates were exposed to increasing concentrations of manganese that often exceeded the natural background levels of Mn in soils.

Metals accumulate in the gastropod soft tissues, especially in the hepatopancreas/digestive glands [13], but there is evidence that some of them (e.g., Pb, Zn) may accumulate in the shell as well [14], [15]. The use of the hepatopancreas of adult snails, instead of whole animal bodies, is therefore suggested for monitoring purposes, particularly in less polluted environments [16], [17]. Terrestrial gastropods are able to accumulate metals directly from soils, independently of food ingestion and in a dose-dependent manner [18]. The direct uptake of metals from soils to snails occurs both via cutaneous contact and soil ingestion [13], [19], [20]. Such mechanisms have been reported for non-essential metals, such as cadmium or lead [21]–[23], as well as for essential metals, such as copper and zinc [18], [24]. This attested to land snails the potential to serve as reliable bioindicators of soil quality, and therefore, standardized toxicity tests applicable to the assessment of contaminated land have also been developed for terrestrial snails (e.g., ISO-15952∶2006).

Manganese was shown to interfere with calcium metabolism in gastropods by breaking down the calcium granules in the hepatopancreas/digestive glands [25] and to induce cellular alterations in the snail soft tissues [26]. This essential metal is transferred along the soil-plant-snail food chains [27], [28], but, the link between soil Mn and land snails has never been been made although these mollusks routinely come in contact with the upper soil horizons through cutaneous contact, egg-laying, and feeding activities in soil [29]–[31]. The present study proposes a realistic approach for filling this gap: the work is conducted under semi-realistic field conditions, with most experimental parameters being similar to those encountered in nature (i.e., temperature, photoperiod, sunlight, air currents). Our purpose in this paper is to address the following practical questions (1) Is manganese transferred directly from soil to snails independently of food ingestion? (2) What is the toxic potential of soil enrichment with Mn on land snails (*Pulmonata*)? To this end, juvenile *Cantareus aspersus* snails were exposed continuously, over 30 and 60 days, to soils spiked with low Mn concentrations. The uptake of manganese from soils to snails was assessed by measuring the metal concentrations in the hepatopancreas, foot, and shell. The snail survival and shell growth were used as accumulation end-points to assess the lethal and sublethal effects of manganese exposure. We considered the scenario in which the soil Mn concentration lies within the natural background level, that is from 40 to 900 milligrams per kilogram dry weight (mg/Kg d. wt) [32]. The results of this study are important, because in anthropized areas the land snails are regularly exposed to anthropogenic releases of manganese, and the direct ingestion of soil plays an important role for the gastropod growth and development [33], [34].

## Materials and Methods

### Ethical Statement

The present study was carried out on private land (45.8747° lat. N, 22.2117° long. E, 85 Temeresti, 305300 Faget, Romania) with the approval of the land owner (Stanila Doina). We mentione that no specific permissions was required to perform the present experiments at this location. Ethical approval is not required for research work with *Cantareus aspersus*. However, this study was performed in accordance with the internal guidelines of Banat’s University of Agricultural Sciences and Veterinary Medicine from Timisoara, Romania. These guidelines comply with the national and European recommendations concerning the protection and welfare of laboratory animals. The purchase of *C. aspersus* for this study did not involve endangered or protected species.

### Experimental Design and Snail Rearing

All the investigations were conducted outdoor in the village of Temeresti (Timis county, Romania; 45.8747° lat. N, 22.2117° long. E). *Cantareus aspersus* (*syn*. *Cornu aspersum* or *Helix aspersa*) was considered in this study because this species is easily reared both under controlled and field conditions [21]–[23], and has been shown to accumulate high amounts of metals in its body [15]. Six weeks prior to the experiment (late April 2011), 1,050 juvenile snails, aged three months, were purchased from a snail farm in Romania (S.C. Bright Group S.R.L., Floresti, Mehedinti county). The snails were housed in polypropylene terrariums during the pre-exposure period, in groups of 70 individuals per each terrarium. In the present experiment, a dark brown chernozem soil, with silty clay loam texture, pH of 6.8, and 7.5% organic carbon content was purchased from a specialized provider and used as a substrate. Each terrarium measured 0.50×1.00×0.40 m and contained a 15 cm-deep layer of flower soil (mean weight: 400±15.44 g). The snails were fed ad libitum on specialized fodder, which contained fodder chalk (15%), wheat meal (10%), corn meal (7%), soya grits (10%), corn germ grits (10%), sunflower grits (15%), monocalcic phosphate (3%), Inlavit (10%), wheat pollard (10%), and vitamin-mineral premix for piglets (10%).

The experimental area (5.00×2.50 m) was protected with a sun shade, and therefore, most experimental parameters were similar to those encountered in nature, i.e., temperature, photoperiod, sunlight, and air currents. The relative air humidity and soil moisture in the environment were recorded two times a day throughout the experiment (5.00 am and 23.00 pm, respectively), and adjusted accordingly within terrariums by using a dew generator and a pressure sprayer. To prevent unintentional contamination of experimental samples we used only double distilled water to generate dew and adjust soil humidity. The fodder was placed in a 7-cm-diameter Petri dish for limiting food contamination through direct contact with the soil. The daily activity schedule consisted of monitoring snail fitness, removal of feces and uneaten food, fresh fodder supply, removal of snails from the terrarium walls and Petri dishes, and collecting dead specimens. The terrariums were cleaned once a week with double distilled water.

Our study focused mainly on Mn transfer from soils to snails as it occurs in natural environments (i.e., mixed exposure via epithelial contact and soil ingestion). The experiment consisted of two distinct, but successive phases during which time the snails were maintained on uncontaminated food in plastic terrariums. The snails were exposed first, for a period of 30 days, to a series of soil Mn concentrations (i.e., the E1 phase), and then, for a second period of 30 days, to soils with higher Mn concentrations (i.e., the E2 phase). The snails were exposed to soils spiked with different levels of manganese chloride, as follows: (1) in the E1 phase: M1, reference group; Mn1.1., 23 mg/Kg d. wt manganese; Mn2.1., 46 mg/Kg d. wt manganese; Mn3.1., 115 mg/Kg d. wt manganese; Mn4.1, 230 mg/Kg d. wt manganese; (2) in the E2 phase: M2, reference group; Mn1.2., 46 mg/Kg d. wt manganese; Mn2.2., 92 mg/Kg d. wt manganese; Mn3.2., 230 mg/Kg d. wt manganese; Mn4.2., 460 mg/Kg d. wt manganese. The rationale of applying two successive exposure periods was (1) to assess whether a new exposure event influences the kinetics of manganese retention in land snails previously exposed to manganese-spiked soils (2) to improve the chance of observing the direct transfer of manganese from soils to snails having different exposure regimes applied to individuals of unequal age and size. Each phase of our experiment lasted 30 days, which is very close to that advised in the ISO-15952 standard for assessing the effects of soil pollutants on the growth and survival of juvenile land snails (*C. aspersus* ), i.e. 28 days.

The toxicity of manganese was investigated under static exposure (i.e., substrate without renewal) for each experimental phase. Before spiking, the substrate was sieved through a 5-mm mesh sieve to remove coarse materials (roots, rocks, macro-organic matter) and particles larger than 5 mm. Manganese chloride is routinely used in industrial and agricultural activities [2] and Mn^+2^ ions are the most soluble species in soil [9]. As a result, pure manganese chloride tetrahidrate (MnCl_2_ 4H_2_O, 99.99% trace metal basis) was purchased from Sigma-Aldrich Chemie GmbH (Buchs, Switzerland) and used to prepare the corresponding spiking solutions. The spiking-solution concentrations were chosen so that Mn concentrations in the spiked substrates lied within the natural background levels in soil (40–900 mg/Kg d. wt). After spiking, the substrate was homogenized in batches in a Waring blender (about 1,200 g per each Mn treatment) and followed an equilibration period for one week prior to each experimental phase. The soil pH was measured at the start of the experiment and at the end of both experimental phases by using a digital pH- meter.

At the start of E1 phase (early June 2011) the snails were sorted based on their size. The shell height (SH) was used to estimate the snail size, because the snail weight is highly variable in terrestrial gastropods [35], whereas the shell growth is irreversible [36]. The shells were also measured at the end of each experimental phase to assess the effects of Mn exposure on shell growth. The measurement method was compiled from the malacological literature [37]. The shell height was assessed with a digital caliper to the nearest 0.001 mm. Every measurement was repeated three times and only the mean value was taken into account. The selected snails, i.e., 750 individuals of similar size with a mean shell height of 1.877 cm, were distributed in five samples of equal size (150 snails per bulk sample), one sample for each manganese treatment. Three replicates of 50 *C. aspersus* juveniles each were used per concentration to reduce the chance of statistical error. The snails were numbered with black acrylic paint to allow a proper identification of each specimen.

For each replicate jar, five topsoil samples (5 g of soil per sample) were collected one day after the beginning of the E1 phase. They were dried (22°C, 7 d), disaggregated, homogenized, sieved to 2 mm, and then stored in self-sealing sterile paper pouches for further analysis. The daily activity schedule was similar to that described during the pre-exposure period.

At the end of E1 phase, 20 snails per each replicate jar were starved for 48 h, and then sacrificed by freezing (−20°C, 4 h). After thawing, the soft body was removed from the shell using a hemostat. The hepatopancreas and the foot were separated, washed in double distilled water, and stored until processing in the lab (chest freezer at −20°C). The empty shells were rinsed three times with sterile double distilled water, dried with sterile paper towels, and kept in self-sealing sterile paper pouches at ambient temperature (22°C). From the snail soft tissues only the hepatopancreas (excluding kidney, heart, gonad, and albumen gland) and the foot (foot/head complex) were investigated in our study because these organs are known to concentrate significant amounts of Mn [27], whereas the shell may serve as the final sink for metals [15]. The same experimental protocol was followed in E2 phase. At the end of the experiment, the investigated snails were still sub-adults.

### Chemical Analyses

The concentrations of Mn in samples (soil, snail shell, snail foot, snail hepatopancreas) were assessed in the Environmental Research Test Laboratory (Banat’s University of Agricultural Sciences and Veterinary Medicine from Timisoara, Romania). All samples were weighed before the chemical analyses on an analytical balance to the nearest 0.0001 mg. The snail foot and hepatopancreas samples were defrosted, oven dried (105°C, 24 h), and calcinated in a muffle furnace (550°C, 4 h). The calcinated shells were entirely disolved in 10 ml of HNO_3_ suprapure (Merck, 65% suprapure), and brought to 45 ml with 35 ml of double distilled water. In the case of soft tissues (i.e., hepatopancreas, foot), the ash was dissolved in 10 ml of 0.5 N HNO_3_ and filtered through ash-free filter paper. The volume was brought to 45 ml with 35 ml of 0.5 N HNO_3_. Manganese was extracted from soil to solution by wet extraction. The samples were digested in 50 ml of 0.5 N HNO_3_ at a ratio of 1∶10 soil and nitric acid solution (22°C, 24 h), and then filtered through ash-free filter paper. For each sample, 10 ml of filtrate were transferred in a sterile polyethylene tube and brought up to 45 ml with 35 ml of 0.5 N HNO_3_.

To assess Mn concentrations in the filtrate we used flame atomic absorption spectrophotometry with high resolution continuum source (Model ContrAA 300, Analytik Jena, Germany). The results were expressed as milligram per kilogram dry weight (mg/Kg d. wt). Mix standard solutions (1000 mg/l) of Mn- ICP multielement standard solution IV CertiPUR, were purchased from Merck. Double distilled water (spectroscopic pure) was used for the preparation of reagents and standards. All glassware was treated with Pierce solution 20% (v/v), rinsed with cold tap water, washed with 20% (v/v) nitric acid, and then rinsed again with double distilled water. For quality control purposes all blanks and duplicate samples were analyzed during the procedure. NCS Certified Reference Material-DC 85104a and 85105a (China National Analysis Center for Iron&Steel) was analyzed for quality assurance. The percent recovery mean was 94%. The variation coefficient was below 10%. Mn detection limit (mg/Kg d. wt), as assessed by using the calibration curve method, was 0.18. The blank reagent and standard reference soil were included into each sample batch to verify the accuracy and precision of the digestion procedure.

### Statistical Analysis

The Mn concentrations in samples and shell height (SH) were tested for normality with an Anderson-Darling test, and for homoscedasticity by using a Levene’s test. Differences among treatments for Mn content in the soil, snail hepatopancreas, snail foot, and snail shell were assessed using a one-way analysis of variance (Anova), with a post hoc Dunnet’s test. For each experimental phase, a simple regression analysis was conducted to investigate relationships between Mn concentrations in the soil and those measured in either the hepatopancreas, foot, or shell. The organs for which the soil-to-organ regressions were statistically significant during both experimental phases were considered as potential biomarkers of Mn accumulation in the soil. A one way analysis of covariance (Ancova) was then conducted to distinguish between the subsequent kinetics of Mn accumulation. If significant differences were observed, a regression analysis was used to determine the effect of a new exposure event on the kinetic of Mn accumulation in the organs of snails previously exposed to Mn-spiked soils (excluding the control snails). The ratio between Mn concentrations in the soil at the end of the E2 and E1 phase was considered as an independent variable, whereas the corresponding ratio in organs was regarded as a dependent variable. Managese levels in the organs of the control snails at the end of the E2 and E1 phase were then compared by using a Mann-Whitney test.

A one-way analysis of variance (Anova) with a post hoc Dunnet’s test was further run to determine the impact of Mn exposure on shell growth (day 0, 30, and 60). For each experimental phase, survival analysis was performed by using a Log-Rank test, followed by Breslow’s tests. A Kaplan-Meier curve was generated from lifetime data to represent the proportion of the snails surviving at successive times for each Mn treatment. The treatment was considered complete for the dead specimens and censored for the snails that were still alive at the end of the study. The mean survival time was assessed as the area under the Kaplan-Meier estimate of the survival curve [38]. The survival rates and mean survival times in the E2 phase refer only to individuals that were not sacrificied at the end of the E1 phase and were alive at the start of the E2 phase.Statistical analysis was performed by using Statistica 10 software package (Statsoft Inc.). A *p* value <0.05 was considered significant.

## Results

### Manganese Accumulation

**Figure 1:**
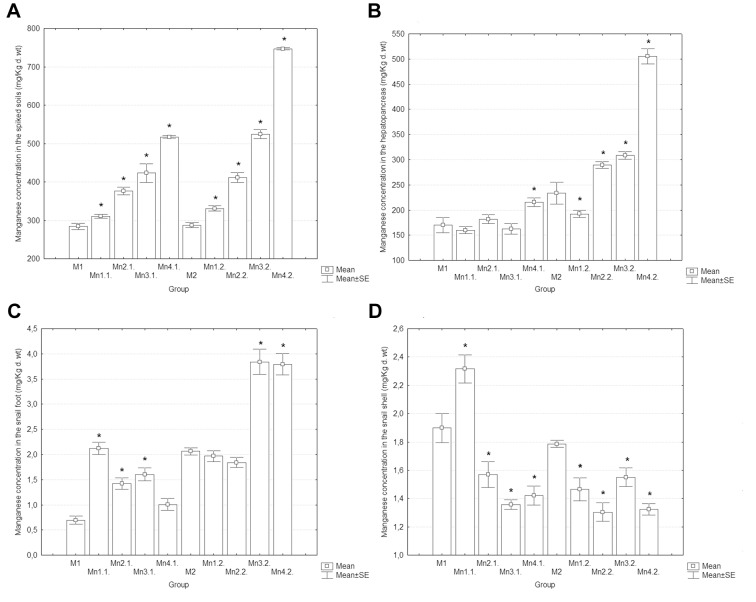
Mean (with SE) manganese concentrations (mg/Kg d. wt). A) soil; B) snail hepatopancreas; C) foot; D) shell. Marked boxes (*) indicate significant differences as compared to the reference group of each experimental phase (Duncan’s test, p<0.05).

The soil pH was constant during the experiment (pH = 6.8). The time for the snails detaching from the substrate while feeding on the fodder in the Petri dishes was about 2–3 hours. The total manganese content of the soil (Figure 1A) increased with the nominal concentration of Mn-spiking solutions (Anova, *p* = 0.000). These results showed that the soil spiking was properly performed, thus validating the experimental premises. There was a consistent effect of soil Mn on concentrations in the hepatopancreas, foot, and shell, irrespective of experimental phase (Anova, *p*<0.003). Increasing manganese concentration in the soil induced different kinetics of accumulation in the investigated organs: Mn concentrations in the hepatopancreas increased (Figure 1B), Mn levels in the foot of the snail remained relatively constant (Figure 1C), whereas Mn concentrations in the shell slightly decreased (Figure 1D).

**Figure 2:**
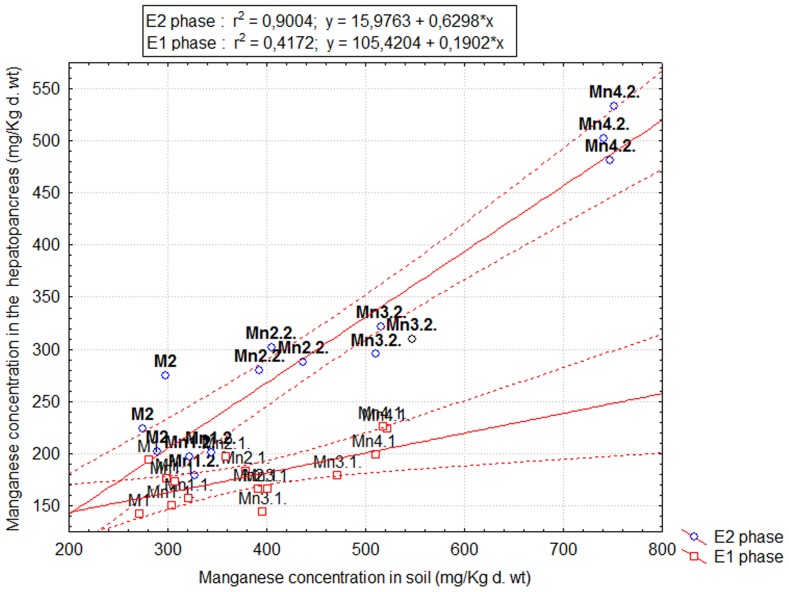
The soil-to-hepatopancreas regressions in the E1 and E2 phases. The scatter plots correspond to the mean manganese concentrations in the soil and hepatopancreas (triplicate determinations).

Manganese was found at much higher concentrations in the snail hepatopancreas than in the foot and shell. This shows that the hepatopancreas is the main storage site for Mn in the snail body. Results of regression analysis showed that, in the E1 phase, the soil Mn concentration was significantly associated with its retention in the hepatopancreas (*p = *0.011, *R^2^* = 0.401) and shell (*p* = 0.015, *R^2^* = 0.374), but not with the level to which this metal accumulates in the foot (*p = *0.802, *R^2^* = 0.002). In the E2 phase, the soil-to-organ regressions were highly significant (hepatopancreas: *p = *0.000, *R^2^* = 0.854; foot: *p = *0.000, *R^2^* = 0.700), excepting the shell (*p = *0.069, *R^2^* = 0.246). Interestingly, the soil-to-hepatopancreas regressions were not similar in the E1 and E2 phase (Figure 2) and we found significant differences between the corresponding slopes (Ancova, *p* = 0.000). The increase in soil Mn content resulting from a new exposure event accounted for a significant proportion of Mn variation in the hepatopancreas of snails previously exposed to Mn-spiked soils (*p* = 0.000, *R^2^* = 0.729). There were also significant differences between Mn concentrations in the hepatopancreas of control snails in the E1 and E2 phase (Mann-Whitney test, *p* = 0.049). Therefore, the accumulation kinetics of Mn in the snail hepatopancreas appears to be related not only to the soil Mn concentrations, but also to other contributing factors.

### Manganese Toxicity

**Figure 3:**
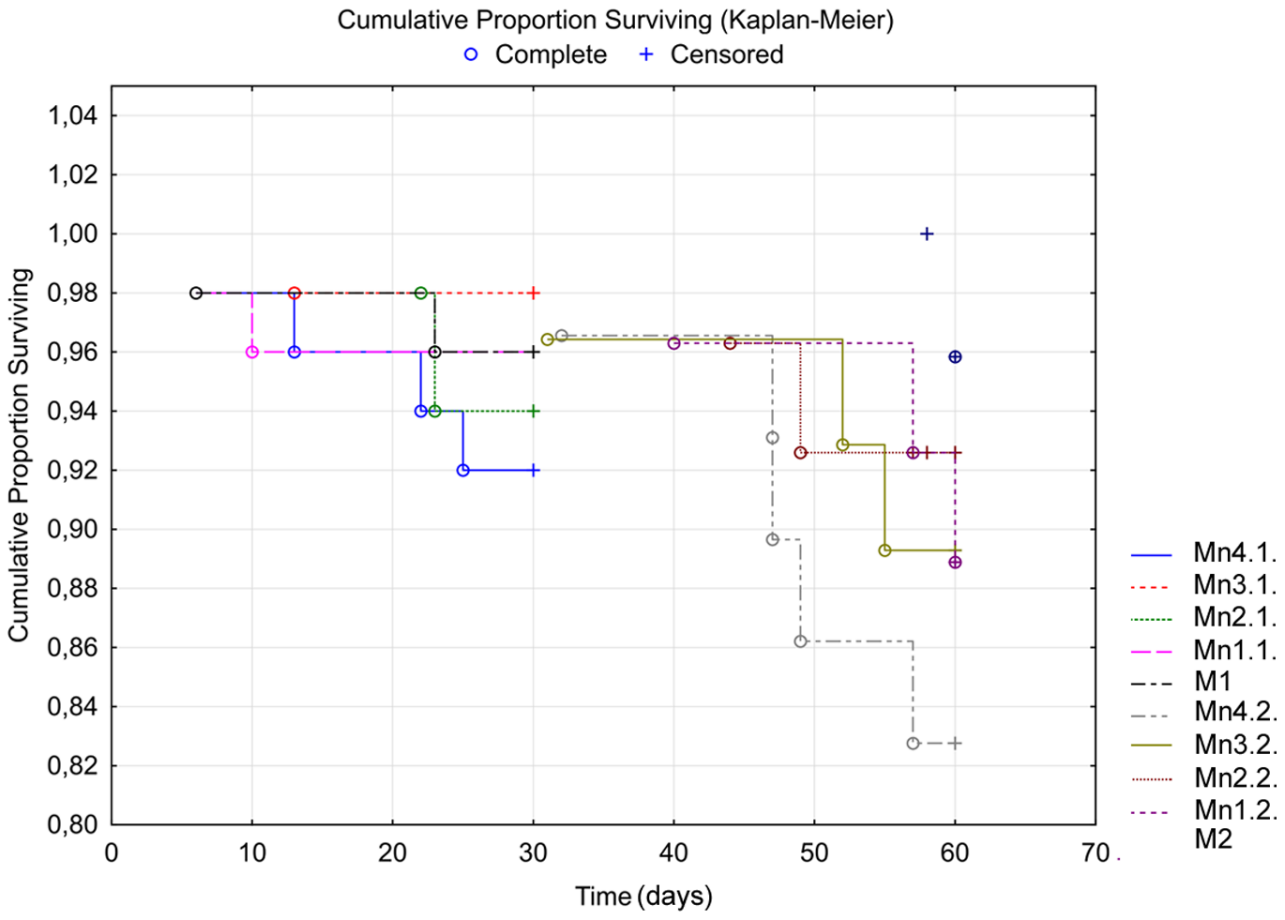
Kaplan*-*Meier survival curves in the E1 phase (left) and E2 phase (right). The complete data relates to the death snails, whereas the censored data are associated with the living individuals.

The results revealed no significant differences for shell height among groups (Table 1) at the beginning of the experiment (Anova, *p* = 0.557), as well as at the end of both experimental phases (Anova, E1 phase: *p* = 0.283; E2 phase: *p* = 0.166). The overall survival in the E1 phase was similar for all metal treatments (Table 2) and the corresponding Kaplan-Meyer survival curves (Figure 3) were not significantly different (Log-Rank test, *p* = 0.657). During the E2 phase, survival percentage decreased as compared to the E1 phase, except for the M2 snails (Table 1b), and the survival curves showed significant differences among treatments (Log-Rank test, *p* = 0.024). Thus, post hoc tests showed that the M2 snails have significantly higher survival rates than either the Mn4.2. snails (Breslow’s test, *p* = 0.003) or the Mn3.2. snails (Breslow’s test, *p* = 0.002), but do not show significant differences in mortalities when compared with either the Mn2.2. snails (Breslow’s test, *p* = 0.062) or the Mn1.2. snails (Breslow’s test, *p* = 0.077).

**Table 1:** Mean (and SE) for shell height in snails exposed to Mn-spiked soil.

	Mean height (cm)			
Day 0	Mntreatment	Day 30	Mntreatment	Day 60
1.915 (0.074)	M1	1.993 (0.087)	M2	2.035 (0.056)
1.793 (0.040)	Mn1.1.	1.872 (0.033)	Mn1.2.	1.912 (0.041)
1.857 (0.053)	Mn2.1.	1.915 (0.046)	Mn2.2.	1.953 (0.043)
1.886 (0.039)	Mn3.1.	1.923 (0.034)	Mn3.2.	1.986 (0.031)
1.839 (0.062)	Mn4.1.	1.897 (0.038)	Mn4.2.	1.944 (0.043)

The values were measured at the start of the experiment (day 0), end of E1 phase (day 30), and end of E2 phase (day 60).

**Table 2:** Snail survival in percentage and mean survival time (and SE) for each Mn treatment.

	Day 30		Day 60		
Mn treatment	Survival (%)	Mean survival time (days)	Mn treatment	Survival (%)	Mean survival time (days)
M1	96.00%	29.380 (0.492)	M2	100.00%	29.920 (0.078)
Mn1.1.	96.00%	29.120 (0.612)	Mn1.2.	89.29%	28.500 (1.051)
Mn2.1.	94.00%	29.560 (0.246)	Mn2.2.	92.59%	29.000 (0.692)
Mn3.1.	98.00%	29.660 (0.336)	Mn3.2.	88.89%	29.148 (0.730)
Mn4.1.	92.00%	28.920 (0.598)	Mn4.2.	82.76%	27.655 (1.139)

The values were measured at the end of E1 phase (day 30) and E2 phase (day 60).

## Discussion

### Manganese Accumulation

This study provides a new perspective on land snails as important ecological links in terrestrial food webs and contributes to the not so well documented knowledge of manganese bioavailability to soil invertebrates. Despite being transferred along soil-plant-snail food chains [27], [28], [39], there is no information available concerning the uptake of manganese by land snails via cutaneous contact and/or soil ingestion. This study provides, for the first time, solid evidence for the existence of such a mechanism by demonstrating that the transfer of manganese from soils to terrestrial gastropods occurs independently of food ingestion. Because land snails mediate the transfer of manganese from soil to the upper trophic levels (i.e., primary consumers) not only along food chains, but also via additional uptake paths (cutaneous contact and/or soil ingestion), these invertebrates are important for understanding the cycle of manganese in terrestrial ecosystems. The ingestion of food is expected to serve as the main route of Mn exposure [26], but the direct transfer from soils to snails should not be neglected when precisely assessing the impact of anthropogenic Mn releases on soil ecosystems.

The uptake of manganese from soils to snails may occur through both soil ingestion and epithelial contact, as it is the case of other metals. Both cutaneous and digestive routes of exposure are equally efficient in transferring Cd from soil to snails [21], but the soil ingestion is more important for zinc uptake as compared to the exposure via epithelial contact [18]. The experimental design did not allow us to clearly differentiate between these two paths of exposure, but there is some indication that the soil ingestion may also serve as the main route of Mn uptake. Although the snail foot epithelium is the largest wet surface in contact with the soil [40], the Mn content of the foot remained relatively constant throughout the study. In contrast, the hepatopancreas Mn levels increased with metal concentrations in the soil. There is, however, further possibility that Mn bioavailability in the soil was low and thus the potential for accumulation via epithelial contact was low as well.

The gastropod shell is basically made of calcium carbonate [41], but, as shown in our study, it naturally contains small amounts of manganese. Because the shell contains less than 5% of the Mn content in the hepatopancreas, this organ cannot be regarded as a long-term sink for Mn. The internal Mn concentrations are not at equilibrium, thus suggesting that land snails are able to retain in their soft tissues higher amounts of manganese than those found in the present work. The accumulation pattern over the different soft tissues (hepatopancreas vs. foot) is consistent with other studies that showed the preferential deposition of manganese in the snail hepatopancreas [27]. The role of hepatopancreas as the main storage site for Mn in the snail body is thereby confirmed here.

In a previous study, we found Mn accumulations of 32.69 up to 131.32 mg/Kg d. wt in the hepatopancreas of newly matured *H. pomatia* that were sampled from different sites in Banat area (Romania) [28]. A recent field survey reported average Mn concentrations of 200–300 mg/Kg d. wt in the hepatopancreas of adult snails, *C. aspersus* and *Helix pomatia*, originating from a semirural area in northern Italy [27]. Results from another study revealed higher Mn levels in the hepatopancreas of adult *C. aspersus* (ranging from 130 up to 726 mg/Kg d. wt). The snails were caged, fed an uncontaminated diet (carrots), and exposed for four weeks to exhaust gases from vehicular traffic [42]. The Mn content of hepatopancreas in the present experiment generally fall between 150 and 325 mg/Kg d. wt, and only for the highest metal concentrations in the soil it slightly exceed 500 mg/Kg d. wt. These results show that the level to which Mn accumulates in the gastropod body depends on various factors, such as snail species, diet composition, or age.

Having a dose-dependent relationship with Mn concentrations in the soil, the hepatopancreas of sub-adult snails may serve as a more sensitive and targeted biomarker of Mn exposure than the whole snail body, which can detect even low levels of soil Mn enrichment. However, such a relationship may not be valid for long-term exposure of soils to manganese from industrial inputs. Since the spiking period in the present study was short, Mn may be weakly bound to the substrate particles, thus making it more bioavailable to land snails as compared to the aforementioned situation. The soil physico-chemical properties, particularly the pH and the redox potential, were demonstrated to serve as major variables that regulate manganese bioavailability in soils [43]. As a result, future studies must be designed to elucidate not only the importance of exposure duration, but also the impact of soil physico-chemical properties, on the kinetics of Mn transfer from soils to terrestrial gastropods. Importantly, there are no reference values concerning the level to which manganese accumulates in the hepatopancreas; therefore, a necessary step would be to precisely assess these values before using the hepatopancreas as a test end-point for Mn side-effects in soil ecotoxicological studies.

The kinetics of Mn retention in the hepatopancreas is significantly modified in the E2 phase as compared to the E1 phase. Increase in soil Mn concentration resulting from a new exposure event is shown here to be an important factor determining the dynamic of Mn retention in the hepatopancreas of snails previously exposed to Mn-spiked soils. Similar effects were reported for Cd, when single and repeated exposure events via food uptake induced different responses of Cd metallothionein in the land snail *Helix pomatia* [44]. Different levels to which Mn accumulates in the hepatopancreas of control snails in the E1 and E2 phase show that additional factors also influence the kinetic of Mn retention in the hepatopancreas. Both endogenous (changed physiology due to growth/age differences) and exogenous variables (photoperiod, duration of exposure, vapor pressure, temperature, mean rainfall level, soil physico-chemical properties) were shown to affect the body burdens of lead, zinc, and cadmium in land snails [15], [45], [46]. We therefore suggest that our results may be better explained by the additive effect of such factors, rather than by one mechanism alone.

### Manganese Toxicity

We have a poor understanding of the toxicity of soil Mn on land snails. A recent study examined the relationships between the soil moisture, organic matter, cation exchange capacity, pH, calcium, magnesium, zinc, potassium, phosphorus, sulfur, boron, manganese, iron and copper and the land snail abundance and diversity. It was found that Mn concentrations in the soil have a negative association with land snail abundance [47]. Our results suggest that a 60-days exposure to neutral soils enriched with Mn (to levels lying within the natural background level) does not alter the shell growth rate, but may significantly affect the survival of juvenile *C. aspersus* snails if manganese concentrations in the hepatopancreas exceed 300 mg/Kg d. wt. This study showed low mortality rates for Mn-exposed snails, which may be associated with low toxicity of manganese under the present experimental conditions (e.g., soil Mn concentrations, chemical form of the Mn compound, and physico-chemical properties of the substrate). Manganese chloride, which is the source of manganese in this study, is widely used in industry, and importantly in animal feed and agricuture to supply Mn to the growth of animals/plants [43]. Given the central position of land snails in terrestrial ecosystems and their value as bioindicator organisms [15], such findings are important in this context, particularly for achieving sustainable agriculture. For example, one can roughly estimate that Mn enrichment of neutral soils via manure/fertilizer application may be safe on short-term for land snails if background manganese concentrations in the soil fell below 750 mg/Kg d. wt.

The growth and survival rate of young snails (usually *C. aspersus*) are routinely used as toxicological end points for assessing the effects of soil contaminants on land snails. This study provides consistent evidence that such biomarkers of exposure may not be reliable for assessing the toxicity of soil manganese on land snails, especially at low levels of soil enrichment/contamination with manganese. To this end, we suggest that a possibility is to correlate the level to which Mn accumulates in the hepatopancreas with other sublethal parameters of toxicity. The land snail *Helix aspersa maxima* showed a dose-dependent pattern of alteration of the basophilic cells in the hepatopancreas when fed a Mn-enriched diet [26], and therefore, the degree of cellular damage in this organ may be used for this purpose. Several other biomarkers also deserve to be investigated for assessing the impact of soil contamination/enrichment with manganese on land snails. For example, it was found that exposure to other essential metals (Cu, Zn) can decrease 5′-nucleotidase in the hepatopancreas of land snails and also cause DNA damage in this organ [48], [49]. Because such biomarkers of toxicity can describe the effects of chemical agents at genetic level it would be desirable to identify similar biomarkers for manganese.

## Conclusions

This study shows, for the first time, that the transfer of manganese from soils to land snails occurs independently of food ingestion. The hepatopancreas serves as the main storage site for manganese, whereas the shell does not function as a long-term sink for this metal. Soil manganese influenced the accumulation of manganese in the hepatopancreas in a dose-dependent manner, but had no consistent effect either on the snail survival or shell growth. Increase in soil Mn concentration resulting from a new exposure event is shown to be an important factor contributing to modification of manganese retention kinetics in the hepatopancreas of snails previously exposed to manganese-spiked soils. In sum, the results of the present experiment attest to the importance of land snails for manganese cycling in terrestrial ecosystems, and suggest that the direct transfer from soils to snails should be considered in environmental hazard assessment of soil manganese enrichment from industrial inputs.
